# Interdisciplinary Collaboration between Natural and Social Sciences – Status and Trends Exemplified in Groundwater Research

**DOI:** 10.1371/journal.pone.0170754

**Published:** 2017-01-27

**Authors:** Roland Barthel, Roman Seidl

**Affiliations:** 1 Department of Earth Sciences, University of Gothenburg, Gothenburg, Sweden; 2 D-USYS Transdisciplinarity Lab, ETH Zurich, Zurich, Switzerland; Universidad Veracruzana, MEXICO

## Abstract

Interdisciplinary collaboration, particularly between natural and social sciences, is perceived as crucial to solving the significant challenges facing humanity. However, despite the need for such collaboration being expressed more frequently and intensely, it remains unclear to what degree such collaboration actually takes place, what trends and developments there are and which actors are involved. Previous studies, often based on bibliometric analysis of large bodies of literature, partly observed an increase in interdisciplinary collaboration in general, but in particular, the collaboration among distant fields was less explored. Other more qualitative studies found that interdisciplinary collaboration, particularly between natural and social scientists was not well developed, and obstacles abounded. To shed some light on the actual status and developments of this collaboration, we performed an analysis based on a sample of articles on groundwater research. We first identified journals and articles therein that potentially combined natural and social science aspects of groundwater research. Next, we analysed the disciplinary composition of their authors’ teams, cited references, titles and keywords, making use of our detailed personal expertise in groundwater research and its interdisciplinary aspects. We combined several indicators developed from this analysis into a final classification of the degree of multidisciplinarity of each article. Covering the period between 1990 and 2014, we found that the overall percentage of multidisciplinary articles was in the low single-digit range, with only slight increases over the past decades. The interdisciplinarity of individuals plays a major role compared to interdisciplinarity involving two or more researchers. If collaboration with natural sciences takes place, social science is represented most often by economists. As a side result, we found that journals publishing multidisciplinary research had lower impact factors on average, and multidisciplinary papers were cited much less than mono-disciplinary ones.

## 1 Introduction

### 1.1 Background and motivation

“*To solve the grand challenges facing society—energy*, *water*, *climate*, *food*, *health scientists and social scientists must work together*”. This statement begins the editorial in the recently published special issue on interdisciplinarity in Nature [1] (p.305). Few scholars will probably disagree with this statement, but specifically, the collaboration between natural and social sciences, which is particularly crucial to tackling the challenges arising from global changes, remains “*a challenging endeavour*” [2] (p.342). In the above-mentioned issue of *Nature*, Ana Viseu reports her experiences as a social scientist working for a natural science research organisation [3]. She concludes that social scientists in collaboration with natural scientists often only play a “service role”, for example, to satisfy the requirements of research funders. This is most probably a subjective view, but considerable literature confirms this impression (e.g. [4, 5]). Social scientists tell us about last-minute invitations to help fulfil funding agencies’ requirement of including societal components in proposals (anecdotal evidence).

In her recent review of the accomplishments of integrating social with physical sciences for water management, Jay Lund states, “*in scholarship*, *physical and social aspects of water management are typically separated and disintegrated*” ([6] (p.5905). Many authors have discussed the reasons for this separation. For example, [7] lists some obstacles to interdisciplinary collaboration between natural and social sciences, which strongly resemble those highlighted 20 years earlier by [8]. However, the growing international consensus on best practices in interdisciplinary research has been pointed out [9], that is, there is knowledge on how to tackle the obstacles.

Nevertheless, there is a lack of knowledge on the actual status of interdisciplinary collaboration between natural and social sciences. If it is the key to overcoming the major challenges of humankind, is it prospering or still neglected and/or poorly performed, despite its obvious importance?

In this study, we have explored these questions by using groundwater research as a case study. We have chosen this relatively narrow field of research primarily due to our detailed knowledge of it. Our assumption is that such detailed knowledge is required to identify research that is potentially interdisciplinary or multidisciplinary. Second, water in general and groundwater in particular represent excellent candidates for interdisciplinary activities. An overwhelming consensus among scientists and practitioners in the water sector is that the pressing problems in water resources management can only be solved in an interdisciplinary way, specifically by the collaboration between natural and social sciences [10–14]. Groundwater comprises 97% of the world’s usable fresh water resources [15]. About 3–4 billion people rely on sufficient and clean groundwater for drinking on a daily basis [16]. The scientific aspects of groundwater range from purely physical, chemical and biological ones to economic, political, social and cultural dimensions. Where, if not here, should interdisciplinarity flourish?

**Fig 1:**
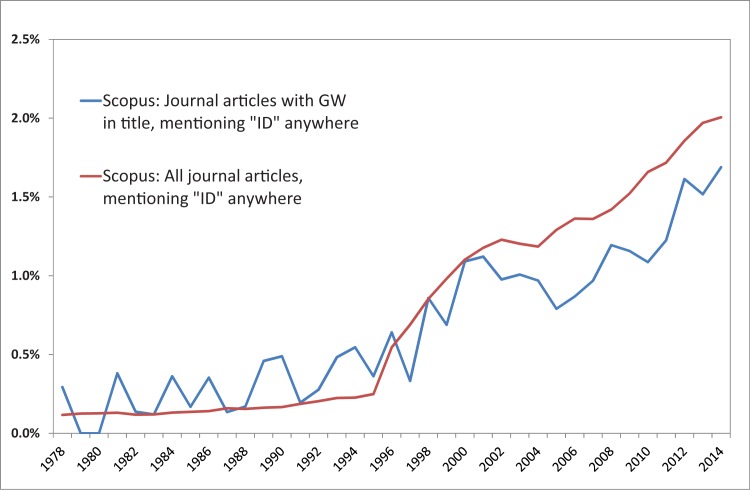
Percentage of journal articles listed in Scopus that mention the term “interdisciplinary” (and similar word forms) anywhere compared to percentage of journal articles with “groundwater” in their titles that mention the term “interdisciplinary” anywhere. GW: “groundwater” or “ground water”-

It has been claimed that research in general is becoming more interdisciplinary, but as [17] point out, the evidence might be anecdotal or partial. In any case, “interdisciplinarity” is increasingly mentioned. Fig 1 shows the development of interdisciplinary research by illustrating the percentage of journal articles mentioning the term “interdisciplinary” (and close word forms) as listed in the Scopus database (www.scopus.com). The strong increase in occurrences of the terms since around 1995 is striking. Fig 1 also shows the results of the same analysis for those articles with the term “groundwater” (or “ground water”) in their titles, abstracts or keywords (the full search strings of all searches carried out to retrieve the data presented in the diagrams and the tables are listed as supplementary material S1 Text). This may be used as evidence that groundwater research does not fundamentally differ from research in general. However, as the remainder of this article shows, this superficial analysis cannot provide proof of rapidly increasing interdisciplinary collaboration in a sub-discipline or a research area.

### 1.2 Terminology

#### 1.2.1 Disciplines, natural and social sciences

Measuring the degree of collaboration between two scientific disciplines requires a clear distinction between them. In the case of natural and social sciences, this may seem relatively straightforward, yet the problems start when examining the details and overlapping areas. For example, overlaps exist in the large and important field of geography where some but not all countries distinguish between physical and human geography and in some sub-fields the division between natural and social sciences are hard to detect. The same is true for certain fields of environmental psychology, or planning and management, all of which combine natural and societal aspects in many different ways. For the purpose of this study, we have found that it is neither possible nor necessary to provide a detailed distinction of the facets of geography, for instance. More generally, disciplinary classifications are not very helpful in the attempt to evaluate interdisciplinary collaboration. This may sound contradictory and is explained later on.

However, a few clarifications of how we define disciplines in this article are required. Social sciences are termed “social” because they deal with humans, their values, preferences, motivations, perceptions, rationales and decisions, from the individual to the collective (societal) level. We follow a simplistic approach here in referring to social sciences; every discipline that does not belong to the natural sciences or engineering is social science. This way, economics (and all of its subspecialisations), administration, law and psychology, as well as arts and humanities, are here combined into social sciences, with the awareness that some economists or psychologists would object. The main reason for doing so is that we have found that the collaboration of natural sciences with “the rest” is limited to an extent and that a further subdivision does not make sense.

Another clarification refers to our usage of the terms “science” and “natural science”. In English-speaking countries, “science” is often used for what other European languages call natural sciences (*Naturwissenschaften*, *les sciences naturelles*, *ciencias naturales*, etc.). To avoid misunderstandings, we always use the term “natural sciences” when referring to disciplines that are not social sciences.

#### 1.2.2 Forms of collaboration among disciplines

Collaborative research encompasses different degrees of cooperation, using the analogy of the ladder of participation in participatory or action research [18]. Since different forms of collaboration among various disciplines are distinguished in the analysis (Section 2.2), some basic definitions of the terms used in this article are provided, as follows:

**Cross-disciplinary (CD)** describes the loosest and least specific form of collaboration among disciplines, “involving or linking two or more scientific disciplines” typically, without a closer description of the purpose and the methodology the involvement.

**Multidisciplinary** is more intense than CD in the sense that multidisciplinary research has a clear purpose of *joint problem solving* by involving different disciplines. However, individual disciplines can work on different aspects of a problem in parallel. Multidisciplinary research is more often temporary, limited to a specific project or problem than interdisciplinary research.

**Interdisciplinary** entails the most intense collaboration, involving the dissolution of disciplinary boundaries, from the problem definition to the methodology. Our understanding of interdisciplinarity follows the definition by Repko et al., who reviews several widely used definitions of interdisciplinary research, extracts their common elements and finally condenses them into the following: “*Interdisciplinary studies is a process of answering a question*, *solving a problem or addressing a topic that is too broad or complex to be dealt with adequately by a single discipline*, *and draws on the disciplines with the goal of integrating their insight to construct a more comprehensive understanding*” [19] (p.25).

**Transdisciplinary** is defined as the cooperation between scientists in academia, on one hand, and practitioners, decision makers or the public at large, on the other hand, which is not examined in this study. Nonetheless, it is important to provide our definition of the term to distinguish it from the others mentioned above, particularly as it is used synonymously with interdisciplinarity or action research [20]. In the European tradition, transdisciplinary “*research takes up concrete problems of society and works out solutions through cooperation between actors and scientists*” [21] (p.6).

### 1.3 Brief overview of methods used to measure interdisciplinary research

Wagner and colleagues [22] reviewed approaches to detect and measure interdisciplinarity in organisations, specific fields of science, countries and so on. They identified a wide variety of approaches, from bibliometric screening of huge bodies of literature using cross-citation analysis and citation mapping to the detailed study of individual organisations or researchers by analysing their structures, approaches and outputs with a combination of different methods (e.g., semi-structured interviews). The most widely used indicators are derived from bibliometric analysis. Journal and article classifications into subject areas or categories as provided by the Web of Science (WoS) or Scopus form the most important source of data for bibliometric analysis [23–25]. By creating maps of cited and citing articles from different disciplines, it can be analysed how closely different disciplines interact (see e.g., [23]). The underlying assumption is that an article that cites others from one or more disciplines than its own and/or an article that is cited by those from other disciplines indicates collaboration across disciplinary boundaries. The possibilities for such an analysis are endless and described in the literature on bibliometrics, scientometrics or informatics (e.g., [26,27,28]). In this study, our focus is not on bibliometric analysis. Other authors have explored and described this field extensively and may have reached the limits in terms of measuring interdisciplinarity.

### 1.4 Objectives and scope

The objective of this study is to determine, as quantitatively as possible, the status and development of the collaboration between natural and social scientists over the last few decades. In contrast to other studies that measured interdisciplinarity by using bibliographic analysis on large bodies of literature, we performed a detailed analysis of a relatively small sample of 203 articles in the field of groundwater research published in 12 journals. We only searched peer-reviewed journal articles despite personal experience and anecdotal evidence indicating that interdisciplinary collaboration may take place more often in work environments that produce “grey literature”.

While we strive to evaluate *interdisciplinary* research, the approaches used in this study are more likely to reveal *multidisciplinary* research. For this reason, we mainly use the term multidisciplinary research when describing the methodology and results and interdisciplinary research only in specific instances.

## 2 Materials and Methods

### 2.1 Data

The data used for this analysis was retrieved from the WoS (https://apps.webofknowledge.com/) and Scopus (https://www.scopus.com/) databases, using their respective web interfaces. For the core of the results, we mainly relied on Scopus, using WoS for cross-checks.

The smallest retrievable entity from the databases comprises individual publications (“sources”). Both the content and related metadata of each publication can be used for analysis, based on individual publications or aggregated groups of publications. Numerical indicators can be retrieved from statistical analysis of metadata and text contents for both grouped and individual articles. Additional sources, such as author curricula vitae (CVs), journal descriptions, homepages of projects and organisations and finally, our own judgement, provided important information.

For the main analysis, articles were aggregated in intervals covering five years each (2010–2014, 2000–2004, etc.); 2014 was used as the upper limit because many articles from 2015 were still in press, and some were not yet listed in the databases when the investigation was carried out (spring of 2016). We chose five-year intervals since individual years did not provide sufficiently large numbers of articles, while decades would have contained too many with regard to the feasibility of the analysis. In the results section, we almost exclusively present results from the three latest 5-year intervals (1990–1994, 2000–2004, 2010–2014) as the earlier intervals (1960, 1970) do contain too few articles.

### 2.2 Methods

#### 2.2.1 Main assumptions

To attain the objective of a quantitative, reliable but technically feasible assessment, we used a combination of indicators that were derived based on the following assumptions:

- A multidisciplinary article is more likely to be written by a team of authors from both natural and social sciences than by a mono-disciplinary team or a single author.
- A multidisciplinary journal article is likely to cite references from both natural and social sciences.
- The keywords and the titles provided by the authors of multidisciplinary articles will contain terms that indicate mixed content from natural and social sciences.

#### 2.2.2 General workflow

**Fig 2:**
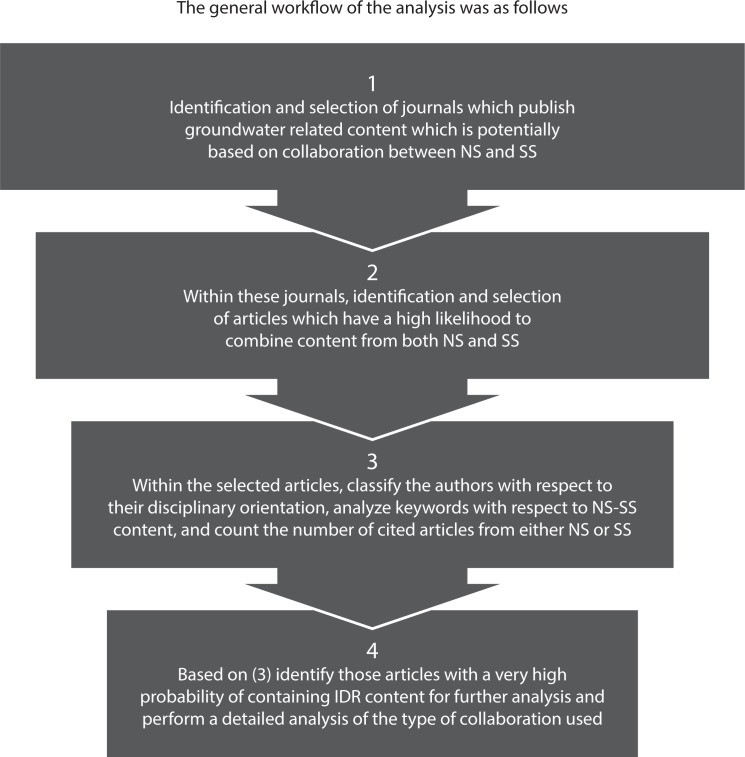
The general workflow of the analysis. NS: natural sciences, SS: social sciences.

Fig 2 depicts the intended workflow used in this study to identify representative journals and their potentially multidisciplinary articles published during the three sampling periods. Particularly, steps 1 and 2 were performed iteratively and required many trials.

#### 2.2.3 Selection of journals and articles

In the Scopus database, 1982 individual sources (72% classified as journals) containing documents with the term “groundwater” in their titles are listed for the 2010–2014 period. In total, 284 journals published more than one article with “groundwater” in the titles per year; 10 journals contain over 25 articles with “groundwater” in the titles per year. The latter group of 10 journals exclusively comprises journals from the natural sciences domain. While all of these journals invite contributions from social sciences and articles on interdisciplinary research, the vast majority of articles (>95%) in this category is from natural sciences. From those 10 journals, we chose water resources research (WRR) for detailed analysis because of its aim and scope, high impact factor (IF) and a long and continuous publishing history. *WRR* is a journal that explicitly points out its interdisciplinary character and invites articles from social sciences [13]; http://agupubs.onlinelibrary.wiley.com/agu/journal/10.1002/(ISSN)1944-7973/#).

For more social science-oriented and/or more multidisciplinary journals, it was not possible to locate a single representative journal with a larger number of groundwater articles per year. *Water International* (*WI*) stood out in this category with an average of six articles per year with “groundwater” in the titles published in 2010–2014. Most of the other journals in this category published less than one groundwater article per year in average. To obtain a sample size equivalent to the number of articles in the *WRR*, a group of 11 “social science journals” was selected for detailed analysis (see Section 3.1).

To identify those journal articles containing potentially multidisciplinary, groundwater-related content, we searched the databases for articles with the term “groundwater” (or “ground water”) in their titles and at least one typical “social science keyword” on their keyword lists. For this purpose, a list of 62 typical keywords used in social science articles on groundwater was derived through an analysis of the keywords used in the articles with “groundwater” in their titles and a “social science” subject area classification in Scopus. Examples from the list of 62 keywords included “stakeholder”, “actor”, “policy”, “governance”, “cost-benefit analysis”, “sustainable management”, “water demand”, “risk assessment” and “decision making” (see the supplementary material S1 Text for the full list and search strings used). As groundwater research, despite its societal relevance, is still predominantly a natural science topic, the occurrence of social science-related keywords in an article with “groundwater” in its title is assumed to be an indicator of mixed content and thus of potential collaboration between natural and social sciences. We are aware that some of these keywords are not necessarily the most typical ones used in social sciences. Rather, such keywords indicate relative closeness to social, political, cultural and economic aspects of groundwater.

#### 2.2.4 Analysis of the composition of author teams

The author teams of the selected articles were analysed, based on the evaluation of each author’s disciplinary orientation, which was assessed by using the following criteria:

- affiliation information available in the databases and/or the article itself;
- each author’s publication records and a qualitative assessment based on titles and journals, focusing on first-authored publications, and changes over time; and
- personal homepages, CVs and information on ResearchGate and LinkedIn.

Table 1 shows the categorisation of individual authors.

**Table 1:** Scheme used to classify the individual authors of a publication.

Author category	Explanation
NS*	Authors with a clear background in and focus on NS or engineering
SS**	Authors with a clear background in and focus on SS (see definition in Section 1.2.1)
**Multidisciplinary**	Authors educated or with backgrounds in/foci on both NS and SS
**Unclear**	No disciplinary background identifiable, particularly, young authors and authors from outside the academia with few publications and little information published on the web

* NS: natural scientist

** SS: social scientist.

While assuming in general that economists would belong to social sciences (see Section 1.2.1), we still found it interesting to count economists separately. In most of the results presented, no distinction is made, but in a few cases, a separate examination of this differentiation reveals additional and important insights.

Using the classifications of the individual authors, author teams were then classified (Table 2).

**Table 2:** Scheme used to classify author teams.

Main classification	Explanation (main classification)	Subdivisions	Explanation (subdivision)
**Mono-disciplinary**	All authors belonging to either NS* or SS**	NS or SS	The author teams’ orientation
**Multidisciplinary**	Mix of authors from SS and NS	Balanced	Roughly equal numbers of NS and SS authors
		NS or SS	Field to which the majority of authors belong
**Weak multidisciplinary**	e.g., one MD*** author in otherwise mono-disciplinary team	NS or SS	Field to which the majority of authors belong
**Single authors**		NS or SS	Single authors’ orientation
		MD	
		Unclear	
Unclear****	No classification possible, e.g., because of too many unclear authors	NS, SS, MD or balanced	Tendency of the team based on those authors with identifiable orientation

* NS: natural scientists team/scientists

** SS: social scientists team/scientist

*** MD: multidisciplinary.

**** The overall classification of an author team can be unclear but still have a clear tendency towards natural sciences, social sciences or MD.

#### 2.2.5 Analysis of cited references

The cited references were analysed by “manually” inspecting them. We looked at each reference and assessed to which field (natural or social sciences) it belonged, based on its title and source. We are confident that the vast majority of cited journal articles, books and book chapters can easily be attributed to the natural or social sciences domain by a person with sound knowledge of the field of research. However, cited references from “grey literature” (reports, manuals, data sets, etc.) and (older) non-indexed materials can pose a methodological challenge as it often remains unclear where they belong. We therefore discounted the number of cited grey literature when determining the ratio of cited social science/natural science references. We then classified the composition of cited references (Table 3).

**Table 3:** Scheme used to classify cited references.

Main classification	Subdivisions	Explanation
**Clearly mono-disciplinary**	NS* or SS**	> 95% of cited references belong to either NS or SS
**Mono-disciplinary**	NS or SS	90–95% of cited references belong to either NS or SS
**Weak multidisciplinary**	NS or SS	75–90% of cited references belong to either NS or SS
**Multidisciplinary**	NS or SS	60–75% of cited references belong to either NS or SS
	Balanced	40–60% of cited references belong to either NS or SS

* NS: natural sciences

** SS: social sciences.

#### 2.2.6 Title, and keyword analysis

An article’s title is assumed to be indicative of its content. We thus performed an experience-based assessment of each article’s title. The approach used was thereby fully heuristic, taking advantage of our knowledge of the field. Keywords related to social sciences that were listed in the Scopus database were determined and counted for each analysed article and the percentage of total number of keywords determined.

#### 2.2.7 Combined evaluation and article classification

The classification results obtained from the analyses of titles and keywords, cited references and author teams were combined into an overall classification of each article. This proved to be a methodological challenge due to the large number of potential combinations, particularly those where one or several of the indicators had been classified as “unclear” or “weak”. Moreover, while the vast majority of articles could clearly and undoubtedly be identified as “clearly natural science” or “clearly social science”, uncertainty was greater for those few articles with some indications of multidisciplinarity. Because of the small sample size and the uniqueness of indicator combinations we chose to perform a merely qualitative analysis that we split into the following two steps:

1. In the “brute classification”, we defined only three article categories–natural science research, social science research and multidisciplinary research. Under the natural science and social science categories, we placed all articles that undoubtedly belonged to either one. All articles with even only weak indicators of multidisciplinarity were classified under the multidisciplinary category in the brute classification.
2. In the “fine classification”, we tried to express different degrees of the likelihood of being natural science, social science and multidisciplinary, depending on the strength of the indicators found and their combinations.

Table 4 explains the categories we defined.

**Table 4:** Categories used for the “fine classification” of articles based on all the different indicators.

Category	Explanation
**Clearly natural science or social science**	All indicators indicate natural science or social science
**Most likely natural science or social science**	Most indicators indicate natural science (or social science), while at least one indicator shows weak contributions from social science (or natural science)
**Maybe multidisciplinary**	Several indicators show weak indications of multidisciplinarity, or one indicator shows strong multidisciplinarity, while the others point to mono-disciplinarity.
**Probably multidisciplinary**	Many indicators point to multidisciplinarity, but one is rather weak or unclear.
**Most likely multidisciplinary**	All indicators indicate multidisciplinarity.

#### 2.2.8 Full text analysis

By step 4 of the workflow (Fig 2), we analysed the full-texts of the articles identified as potentially multidisciplinary (see Table 4) with respect to background and the type of collaboration between natural sciences and social sciences. This analysis was performed in a qualitative way, mainly by searching the methodology sections of the articles for descriptions of the interaction between natural and social scientist in the respective studies. Additionally, we performed searches in the text of all analysed articles looking for terms that allow describing the nature of collaboration (word-families inter-, multi-, cross-, transdisciplinary, collaboration, integration etc.).

#### 2.2.9 Scientific impact

For each article, the number of citations received was retrieved from Scopus (citation data from Scopus assessed in August 2016). The average number of citations by the article category (see Table 4) was calculated. Additionally, the journal impact factors for the chosen journals in 2014 were used to calculate the average journal impact factor for each article category (see Table 4). As this analysis was outside the main scope of the study, we did not exert much effort to remove biases. For example, we neither took into account the impact factor changes over time nor considered that older articles had higher numbers of citations.

## 3 Results and Discussion

### 3.1 Journal and article selection

Table 5 shows the 12 journals that were selected according to the criteria described in Section 2.2.3. It is noteworthy that the total number of articles published in the 12 selected journals increased only moderately over time (plus 20% from 1980–1984 to 2010–2014), while almost four times more articles with “groundwater” in their titles and 30 times more articles with “groundwater” in their titles and at least one social science keyword were published. As shown later (Section 3.5), the number of articles dealing with societal issues in relation to groundwater had not increased dramatically, while the number of keywords related to societal issues assigned to the articles had spiked. These findings indicate that authors try to emphasise that their work is related or applicable to societal issues even if such issues are not at all included in their work.

**Table 5:** Journals and their respective numbers of articles matching the criteria “groundwater” in their titles and “social science keywords” on their keyword lists.

Category	Journal	1980–1984			1990–1994			2000–2004			2010–2014		
		All**	GW***	GW&SS****	All	GW	GW&SS	All	GW	GW&SS	All	GW	GW&SS
1*	**Water Resources Research (WRR)**	1004	70	3	1704	179	24	1664	110	17	2726	210	57
2*	**Ambio**	264	0	0	406	1	1	495	1	0	566	6	4
	**American Journal of Agricultural Economics**	670	1	1	699	5	4	552	2	1	525	5	5
	**Applied Geography (AG)**	1992	5	0	220	3	2	90	1	1	799	4	2
	**Ecological Economics**	0	0	0	178	1	1	613	1	1	1333	9	8
	**Journal of Environmental Planning and Management**	0	0	0	77	1	1	234	3	1	379	6	4
	**Journal of Water Resources Planning and Management**	81	4	0	313	20	14	230	13	10	437	10	8
	**Nature, Nature Geosciences, Nature Climate Change**	15699	3	0	15439	3	1	12708	3	0	14217	23	8
	**Science**	8923	7	0	11627	6	1	12823	9	1	11586	5	2
	**Sustainability / Switzerland**	0	0	0	0	0	0	0	0	0	1527	9	5
	**Water International (WI)**	153	1	0	123	1	0	335	20	7	343	31	12
	**Water Policy**	0	0	0	0	0	0	227	5	2	373	11	8
	**All selected Journals**	28786	91	4	30786	220	49	29971	168	41	34811	329	123

* 1: Natural Science Journal, 2: "Social Science" and Multidisciplinary Journals.

** All: All articles published in the respective journal/period.

*** GW: All articles with groundwater in title, published in the respective journal/period.

**** GW&SS: All articles with groundwater in title and at least one “social science keyword” in keywords, published in the respective journal/period.

Some journals (*Water Policy*, *Sustainability* and *Ecological Economics*) did not exist yet in the first two sample time intervals or might have changed their names and/or foci. For example, *WI* hardly published any papers with “groundwater” in their titles before 1998, while there were 10 such articles in 2014 alone. Such biases are unavoidable and partly show the shift from previous perceptions and publication strategies to the current ones. Traditionally “open” journals such as *WRR* have mainstreamed their content to natural science topics, probably to gain higher impact, thus forcing the authors interested in published multidisciplinary and social science topics on groundwater to choose other journals. New journals emerge as platforms for those articles.

For the detailed analysis, all articles with “groundwater” in their titles and at least one “social science keyword” on their keyword lists were retrieved from the Scopus database for each selected journal (Table 5). The 1980–1984 period was excluded as too few articles matching the search criteria were published during this time interval. From the 223 articles identified by using these selection criteria 20 were later-on removed because they proved to be short comments, editorials, etc. However, we decided to not exclude reviews and opinion articles. All 203 articles used for the evaluation as well as the individual results of the analysis are listed in the supplementary material S1 Spreadsheet.

### 3.2 Composition of author teams

The analysis of author teams was performed according to the principles described in Section 2.2.4. As expected, the classification of authors and author teams proved difficult in some cases, and the results could be ambiguous regarding authors who obtained degrees in different disciplines, changed or broadened their foci during their careers or never really specialised in any field. A major challenge was posed by those authors with backgrounds in and foci on disciplines that were hard to characterise as such. The most prominent of this type comprised geography and disciplines in the field of (environmental) planning and management. A surprising result was the large number of papers written by single authors (32, i.e., 15% of all papers analysed). A substantial number of such papers presented contents that could be rated as multidisciplinary research (12 out of 32, or 38%), but these were not necessarily written by authors we had categorized as multidisciplinary. Table 6 shows the results of the analysis of author teams who wrote the 203 selected articles from the three, five-year periods.

**Table 6:** Results of the classification of author teams (see Table 2 for an explanation of the categories).

Period	Source	single author	mono-disciplinary	multi-disciplinary	weak multi-disciplinary	unclear	Grand Total
**1990–1994**	All*	13 (28.3%)	15 (32.6%)	3 (6.5%)	1 (2.2%)	14 (30.4%)	46
	SS**	9 (40.9%)	4 (18.2%)	2 (9.1%)	1 (4.5%)	6 (27.3%)	22
	WRR***	4 (16.7%)	11 (45.8%)	1 (4.2%)	0 (0%)	8 (33.3%)	24
**2000–2004**	All	5 (12.8%)	23 (59%)	3 (7.7%)	1 (2.6%)	7 (17.9%)	39
	SS	3 (13.6%)	13 (59.1%)	2 (9.1%)	0 (0%)	4 (18.2%)	22
	WRR	2 (11.8%)	10 (58.8%)	1 (5.9%)	1 (5.9%)	3 (17.6%)	17
**2010–2014**	All	14 (11.9%)	76 (64.4%)	12 (10.2%)	6 (5.1%)	10 (8.5%)	118
	SS	11 (17.5%)	29 (46%)	9 (14.3%)	6 (9.5%)	8 (12.7%)	63
	WRR	3 (5.5%)	47 (85.5%)	3 (5.5%)	0 (0%)	2 (3.6%)	55
**Total**		32 (15.8%)	114 (56.2%)	18 (8.9%)	8 (3.9%)	31 (15.3%)	**203**

* All analysed articles from the respective period.

** Articles from the group of 11 “social science” and multidisciplinary journals (see Table 5).

*** Articles from Water Resources Research.

Despite the relatively small sample size, resulting in partly very limited numbers for the individual categories, Table 6 shows some interesting tendencies, as follows:

- The percentage of author teams that cannot be clearly classified decreases over time, partly because the access to information on individual authors has become much easier, but maybe also due to a trend towards more specialisation of individuals.
- The shares of multidisciplinary and weak multidisciplinary author teams have remained rather constant over all periods. The strong increase in strictly mono-disciplinary teams in all categories is foremost a result of the decreasing proportion of unclear authors–again an indication of more specialisation.
- The percentage of single authors has decreased, and the number of authors per paper has roughly doubled from two to four on average (Table 7).

**Table 7:** Results of the classification of individual authors with an unclear, social sciences or multidisciplinary background.

Period	Sources	Average number of Authors	Social science authors	Economists of all authors	Economists of social science authors	Unclear authors	Multi-disciplinary Authors	Number of authors
1990–1994	All*	2.00	29.3%	18.5%	63.0%	20.7%	6.5%	92
	SS**	1.95	27.9%	18.6%	66.7%	20.9%	9.3%	43
	WRR***	2.04	30.6%	18.4%	60.0%	20.4%	4.1%	49
2000–2004	All	2.36	23.9%	14.1%	59.1%	6.5%	13.0%	92
	SS	2.36	21.2%	7.7%	36.4%	1.9%	13.5%	52
	WRR	2.35	27.5%	22.5%	81.8%	12.5%	12.5%	40
2010–2014	All	3.53	19.6%	14.5%	74.1%	4.1%	5.8%	414
	SS	3.24	36.1%	26.7%	74.0%	5.0%	10.9%	202
	WRR	3.85	3.8%	2.8%	75.0%	3.3%	0.9%	212
Total	All	2.96	21.7%	15.1%	69.2%	7.0%	7.0%	598

* All analysed articles from the respective period.

** Articles from the group of 11 “social science” and multidisciplinary journals (see Table 5).

*** Articles from Water Resources Research.

Regardless of the source and the period, 2/3 to 3/4 of all “social science” authors have a background in economics (Table 7), with a slightly increasing number over time. An even closer examination reveals that a large number of articles with a multidisciplinary and/or social science background can be allocated to the “agriculture-economics-groundwater” nexus, yet we have not conducted a quantitative analysis in this direction. The dominance of economic sciences in collaboration between natural and social sciences has been identified by other scholars (e.g., [2, 29]) and has been explained with the quantitative nature of economics, which is more comprehensible to natural scientists than “softer” social science approaches (e.g. [30]).

It should be pointed out that the shown percentages in Table 6 and Table 7 refer to the sample of analysed papers (203), that is, those that were selected because they were identified as potentially multidisciplinary research. In terms of *all* groundwater-related articles published in their respective journals, multidisciplinary teams would have a much lower share.

### 3.3 Cited references

The results concerning cited references are twofold, as follows:

1. We share some general observations regarding journal and article classifications made by Scopus and the WoS. These indicate that a lot of previous work using “unsupervised” bibliometric analysis, i.e., relying on the WoS, such as [31]to measure developments in interdisciplinary research could be misleading.
2. We have classified the cited references according to the principles described in Section 2.2.5

#### 3.3.1 General observations on the analysis of cited references

As described in Section 1.3, the underlying assumption of using bibliometric analysis to measure interdisciplinary research is that an article that cites others from one or more disciplines than its own and/or an article that is cited by papers from other disciplines indicates connections across disciplinary boundaries. This assumption is compelling; however, some fundamental problems associated with its application in practice can lead to severe misinterpretations. To apply those principles, both the citing and the cited articles have to be classified into disciplines. For this purpose, most studies applying citation analysis rely on the classifications used in the WoS or Scopus. However, many authors have already questioned the reliability of journal and article classifications by Scopus and the WoS and have discussed the resulting problems with measurements of interdisciplinary research [25, 32–36]. We provide two examples from our study to illustrate this difficulty.

Example 1. In the WoS, all articles in *WI* are classified as belonging to the *research domain* “science and technology”, the *research areas* “water resources; engineering” and the *WoS categories* “civil engineering or water resources” (similarly in Scopus). All articles in the journal inherit this classification. However, only about 1/3 of the *WI* articles analysed in this study have a natural science focus; all the others are wrongly classified.

Example 2. All articles from *WRR* are classified as belonging to the research domain “science technology” and to research areas of the natural sciences domain (both the WoS and Scopus). Nevertheless, according to this study’s results (reported below), *WRR* used to publish (and to a lesser degree, still does) a significant percentage of papers with social science content. The title “*Cooperative institutions for sustainable common pool resource management*: *Application to groundwater*” ([37] is clearly identifiable as an article from the field of economics, yet the WoS categories for this article are “environmental sciences; limnology; water resources”.

The examples listed in the previous section do not disqualify citation analysis as a tool for measuring multidisciplinary research; they only indicate that the classifications of the WoS and Scopus should be used with great care. Generally, this is a significant drawback for bibliometric analysis as the only reliable alternative is to “manually” classify references. In this context, it is surprising that a large number of studies are concerned with developing new and refining existing approaches to measure connections among scientific disciplines, without making attempts to validate their respective results with “ground truth data”.

With this rationale in mind, we present the results of our reference analysis in the next section.

#### 3.3.2 References in the analysed articles

Table 8 shows the obtained classifications of cited references based on the manual analysis of 8239 individual references. Similar to the number of authors per paper, the number of cited references per paper has almost doubled within the analysed period. With respect to the multidisciplinarity of the cited references, no dramatic changes have occurred over the decades. Examining all analysed journals together, we observe a slight increase in reference lists we classified as multidisciplinary, while in *WRR*, reference lists we would classify as mono-disciplinary have increased, again confirming this journal’s shift in focus to natural sciences.

**Table 8:** Classification of the analysed articles with respect to the cited references according to the scheme described in Table 3.

Period	Source	Clearly mono-disciplinary	Mono-disciplinary	Multi-disciplinary	Weak multi-disciplinary	Total
**1990–1994**	All*	25 (54.3%)	9 (19.6%)	5 (10.9%)	7 (15.2%)	46
	SS**	13 (59.1%)	2 (9.1%)	3 (13.6%)	4 (18.2%)	22
	WRR***	12 (50%)	7 (29.2%)	2 (8.3%)	3 (12.5%)	24
**2000–2004**	All	25 (64.1%)	4 (10.3%)	4 (10.3%)	6 (15.4%)	39
	SS	15 (68.2%)	3 (13.6%)	2 (9.1%)	2 (9.1%)	22
	WRR	10 (58.8%)	1 (5.9%)	2 (11.8%)	4 (23.5%)	17
**2010–2014**	All	72 (61%)	14 (11.9%)	20 (16.9%)	12 (10.2%)	118
	SS	29 (46%)	10 (15.9%)	14 (22.2%)	10 (15.9%)	63
	WRR	43 (78.2%)	4 (7.3%)	6 (10.9%)	2 (3.6%)	55
**Total**		122 (60.1%)	27 (13.3%)	29 (14.3%)	25 (12.3%)	203

* All analysed articles from the respective period.

** Articles from the group of 11 “social science” and multidisciplinary journals (see Table 5).

*** Articles from Water Resources Research.

Interestingly, multidisciplinary articles seem to have a tendency to contain a higher number of citations of “grey” and non-scientific literature (reports, data sets, newspaper articles etc.), which is probably a consequence of their authors being more concerned with their research application to real-world problems and thus more reliant on actual information as provided in statistical reports, maps and so on (Table 9). This confirms other authors’ conclusion that there is a relationship between interdisciplinarity and the degree of concern with applications[38]. A few of the articles analysed, contain hardly any references to journal articles. In those cases, it is very difficult to characterize the disciplinary composition of the reference lists.

**Table 9:** Percentages of references that were excluded from the classification of reference discipline (data sets, reports, legal documents, non-scientific materials) in relation to the final article classifications.

Final article classification	1990–1994	2000–2004	2010–2014	Grand Total
clearly natural sciences	9%	8%	2%	4%
most likely natural sciences	12%	25%	15%	16%
maybe multidisciplinary	27%	10%	26%	24%
probably multidisciplinary	13%	43%	49%	39%
most likely multidisciplinary		22%	13%	14%
most likely social sciences	15%	23%	18%	19%
clearly social sciences	9%	19%	9%	11%
Grand Total	14%	15%	11%	12%

It should be noted, that the analysis of references was carried out with those references listed in Scopus and / or WoS and not on the references printed (or pdf) full texts. While cited journal articles of indexed journals and most books are usually well represented in the databases, information on references from “other” sources may be only partial or missing. We also found that the number of references listed in Scopus or WoS differs from the original number of references in the printed article, and that it may vary over time.

### 3.4 Title and keyword analysis

**Fig 3:**
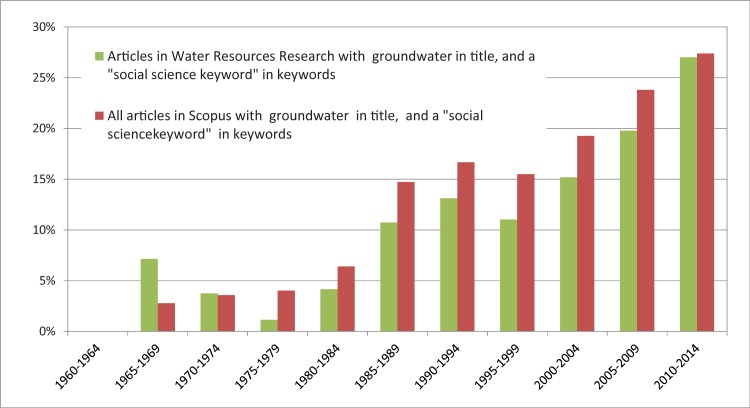
Percentage of articles that have “groundwater” or “ground water” in their title and any of the social science keywords described in section 2.2.3 in the list keywords for Water Resources Research compared to all journal articles listed in Scopus.

The percentage of titles indicating multidisciplinary contents has been decreasing from the first to the last analysed five-year period (Table 10), from 61% to 32% of the analysed articles. This contradicts the results of the keyword analysis, which shows a clear increase of a mix of natural and social science keywords (Fig 3). While in the beginning of the 70^th^, less than 4% of all groundwater-related articles contain any social science keywords, the percentage in the beginning of the 2010s is 30%. Again, WRR started out with a higher than average percentage of social science contents but adjusted to the average later. From these findings, we infer an increasing number of authors’ desire to point out their works’ relation to “societal aspects”, such as management and decision making. At the same time, there is no indication of an equivalently strong increase in works actually dealing with such societal aspects. Unfortunately, it proves that the keywords provided in the databases comprise a mix of author-provided and engineered keywords and that a large number of articles do not contain any author keywords, while others only have author-provided keywords. The ways that engineered keywords are generated by the WoS and Scopus (keyword plus, indexed keywords, etc.) are partly hard to comprehend and do not seem to be well controlled (see also, [39]).

**Table 10:** Assessment of the titles of all analysed articles (heuristic approach).

		Title indicating multidisciplinary content			
Period	Source	no	unclear	yes	Total
1990–1994	All*	17 (37%)	1 (2.2%)	28 (60.9%)	46
	SS**	7 (31.8%)	0 (0%)	15 (68.2%)	22
	WRR***	10 (41.7%)	1 (4.2%)	13 (54.2%)	24
2000–2004	All	19 (48.7%)	1 (2.6%)	19 (48.7%)	39
	SS	9 (40.9%)	1 (4.5%)	12 (54.5%)	22
	WRR	10 (58.8%)	0 (0%)	7 (41.2%)	17
2010–2014	All	77 (65.3%)	3 (2.5%)	38 (32.2%)	118
	SS	37 (58.7%)	3 (4.8%)	23 (36.5%)	63
	WRR	40 (72.7%)	0 (0%)	15 (27.3%)	55
Total		113 (55.7%)	5 (2.5%)	85 (41.9%)	203

* All analysed articles from the respective period.

** Articles from the group of 11 “social science” and multidisciplinary journals (see Table 5).

*** Articles from Water Resources Research.

Table 11 shows the result of the “social science keyword” count and the percentage of such keywords from the total number of keywords. The results are presented in relation to the final article classification for the three analysed periods. As expected, natural science articles contain less social science keywords than social science or multidisciplinary articles. Changes over time are unclear. These results should be interpreted with great care, as only 55% of the articles have author provided keywords, 83% have engineered index keywords. As mentioned above the index keywords are often hard to comprehend.

**Table 11:** Percentages of “social science” related keywords from all keywords in relation to the final article classifications.

	Average percentage of social science keywords			
Final article classification	1990–1994	2000–2004	2010–2014	Grand Total
clearly natural sciences	22%	8%	9%	12%
most likely natural sciences	23%	16%	24%	22%
maybe multidisciplinary	35%	20%	42%	36%
probably multidisciplinary	26%	22%	56%	44%
most likely multidisciplinary		80%	42%	50%
most likely social sciences	34%	52%	51%	49%
clearly social sciences	42%	28%	48%	43%
Grand Total	29%	23%	26%	26%

While performing the qualitative analysis of titles and keywords, we have also tried to identify and classify the main subjects dealt with by the potentially multidisciplinary articles. Because of the low numbers of multidisciplinary articles and the wide range of topics, a crisp classification is not possible. Nevertheless, we have identified the dominance of articles from the “agriculture-economy-groundwater”, the “groundwater-ecosystem services” and the “groundwater-environmental impact assessment” subject areas. Particularly, the dominance of economic aspects is striking and concurs well with the identified prevalence of authors from economic sciences who are involved in multidisciplinary collaboration (Section 3.2, Table 7).

### 3.5 Combined evaluation of indicators

Here, we combine all the individual results (author teams, titles and references) into a final paper classification. We first present the results of the brute classification, where:

1. unclear assessments (e.g., of author teams) were turned into clear results by making assumptions/best guesses and
2. ranges of classifications were combined into one (e.g., multidisciplinary and weak multidisciplinary references into multidisciplinary).

Table 12 shows the results obtained. The brute classification very likely leads to a higher number of articles classified as potentially multidisciplinary. For example, an article with a weak multidisciplinary team and a weak multidisciplinary mix of references would be classified as multidisciplinary research. Using this approach, we have not found any obvious trends in multidisciplinary research over the years.

**Table 12:** Brute classification of the 203 analysed articles into multidisciplinary and mono-disciplinary (natural or social sciences) categories. Numbers in round brackets show percentages of all analysed papers (n = 203). Numbers in square brackets show percentages of all papers with “groundwater” in their titles (n = 717) from the 12 selected journals.

Period	Source	Multidisciplinary articles	Natural sciences articles	Social science articles	Analysed articles	Groundwater articles per source
**1990–1994**	All*	10	26	10	46	220
		(21.7%)	(56.5%)	(21.7%)		
		[4.5%]	[90.9%]	[4.5%]		
	SS**	6	11	5	22	41
		(27.3%)	(50%)	(22.7%)		
		[14.6%]	[73.2%]	[12.2%]		
	WRR***	4	15	5	24	179
		(16.7%)	(62.5%)	(20.8%)		
		[2.2%]	[95%]	[2.8%]		
**2000–2004**	**All**	6	21	12	39	168
		(15.4%)	(53.8%)	(30.8%)		
		[3.6%]	[89.3%]	[7.1%]		
	**SS**	2	14	6	22	58
		(9.1%)	(63.6%)	(27.3%)		
		[3.4%]	[86.2%]	[10.3%]		
	**WRR**	4	7	6	17	110
		(23.5%)	(41.2%)	(35.3%)		
		[3.6%]	[90.9%]	[5.5%]		
**2010–2014**	**All**	21	73	24	118	329
		(17.8%)	(61.9%)	(20.3%)		
		[6.4%]	[86.3%]	[7.3%]		
	**SS**	17	25	21	63	119
		(27%)	(39.7%)	(33.3%)		
		[14.3%]	[68.1%]	[17.6%]		
	**WRR**	4	48	3	55	210
		(7.3%)	(87.3%)	(5.5%)		
		[1.9%]	[96.7%]	[1.4%]		
**Total**	** **	37	120	46	203	717
		(18.2%)	(59.1%)	(22.7%)		
		[5.2%]	[88.4%]	[6.4%]		

* All analysed articles from the respective period.

** Articles from the group of 11 “social science” and multidisciplinary journals (see Table 5).

*** Articles from Water Resources Research.

The results of this brute classification are quite stable from the first to the last analysed period, suggesting that the ratio of natural to social sciences in multidisciplinary papers on groundwater research has not changed significantly. We have therefore attempted using a finer, more differentiated classification scheme. On one hand, this may provide a more realistic picture of multidisciplinary research in the analysed samples. On the other hand, the finer classification leads to very small numbers in some categories, particularly those papers that strongly indicate a multidisciplinary context. Table 13 shows the results. Only five articles (2.5% of the analysed papers and 0.7% of all papers with “groundwater” in their titles in the analysed journals) have been determined as “most likely” multidisciplinary (i.e. all indicators point to multidisciplinarity). This category shows a strong yet most likely insignificant increase from no article in 1990–1994 to four articles in 2010–2014. A weaker increasing trend can be observed for those papers classified as “probably” multidisciplinary, and “maybe multidisciplinary”. It needs to be pointed out, however, that most of the articles where the chosen indicators indicate “maybe multi-disciplinary”, in particular those written by single authors are often mono-disciplinary (see next section). What can be concluded is that the numbers of multi-disciplinary papers are very low, too low to reveal any significant trends.

**Table 13:** Fine classification of the 203 analysed articles. Numbers in **bold print** show percentages of all analysed papers (matching criteria described in Section 2.2.3). Numbers in *italics* show percentages of all papers with “groundwater” in their titles.

Period	Source	clearly natural sciences	most likely NS	clearly social sciences	most likely social sciences	most likely multidisciplinary	probably multidisciplinary	maybe multidisciplinary	All analysed articles	Groundwater articles per source
1990–1994	All*	25	8	6	4	0	2	9	46	220
		**37%**	**17.4%**	**13%**	**8.7%**	**0%**	**4.3%**	**19.6%**		
		*90*.*5%*	*3*.*6%*	*2*.*7%*	*1*.*8%*	*0%*	*0*.*9%*	*4*.*1%*		
	SS**	7	4	3	2	0	2	4	22	41
		**31.8%**	**18.2%**	**13.6%**	**9.1%**	**0%**	**9.1%**	**18.2%**		
		*73*.*2%*	*9*.*8%*	*7*.*3%*	*4*.*9%*	*0%*	*4*.*9%*	*9*.*8%*		
	WRR***	10	4	3	2	0	0	5	24	179
		**41.7%**	**16.7%**	**12.5%**	**8.3%**	**0%**	**0%**	**20.8%**		
		*94*.*4%*	*2*.*2%*	*1*.*7%*	*1*.*1%*	*0%*	*0%*	*2*.*8%*		
2000–2004	All	16	5	4	7	1	1	5	39	168
		**41%**	**12.8%**	**10.3%**	**17.9%**	**2.6%**	**2.6%**	**12.8%**		
		*89*.*3%*	*3%*	*2*.*4%*	*4*.*2%*	*0*.*6%*	*0*.*6%*	*3%*		
	SS	10	4	2	4	1	0	1	22	58
		**45.5%**	**18.2%**	**9.1%**	**18.2%**	**4.5%**	**0%**	**4.5%**		
		*86*.*2%*	*6*.*9%*	*3*.*4%*	*6*.*9%*	*1*.*7%*	*0%*	*1*.*7%*		
	WRR	6	1	2	3	0	1	4	17	110
		**35.3%**	**5.9%**	**11.8%**	**17.6%**	**0%**	**5.9%**	**23.5%**		
		*90*.*9%*	*0*.*9%*	*1*.*8%*	*2*.*7%*	*0%*	*0*.*9%*	*3*.*6%*		
2010–2014	All	59	10	12	13	4	5	15	118	329
		**50%**	**8.5%**	**10.2%**	**11%**	**3.4%**	**4.2%**	**12.7%**		
		*85*.*1%*	*3%*	*3*.*6%*	*4%*	*1*.*2%*	*1*.*5%*	*4*.*6%*		
	SS	15	6	11	13	2	5	11	63	119
		*23*.*8%*	*9*.*5%*	*17*.*5%*	*20*.*6%*	*3*.*2%*	*7*.*9%*	*17*.*5%*		
		64.7%	5%	9.2%	10.9%	1.7%	4.2%	9.2%		
	WRR	44	4	1	0	2	0	4	55	210
		**80%**	**7.3%**	**1.8%**	**0%**	**3.6%**	**0%**	**7.3%**		
		96.7%	1.9%	0.5%	0%	1%	0%	1.9%		
Total		92	23	22	24	5	8	29	203	717
		**45.3%**	**11.3%**	**10.8%**	**11.8%**	**2.5%**	**3.9%**	**14.3%**		
		*87*.*7%*	*3*.*2%*	*3*.*1%*	*3*.*3%*	*0*.*7%*	*1*.*1%*	*4%*		

* All analysed articles from the respective period.

** Articles from the group of 11 “social science” and multidisciplinary journals (see Table 5).

*** Articles from Water Resources Research.

### 3.6 Results of the full-text analysis

Because information on the background and type of the collaboration applied is not sufficiently available in the full-text of the analysed articles, the presentation of analysis results is limited to a summary. Each of the 37 articles which were identified as likely being the result of a multidisciplinary collaboration between natural and social sciences would require an individual discussion and, moreover an analysis of external sources of information. Information on the background and nature of collaboration is hardly mentioned in any of the respective articles. A typical example for the interaction between social and natural sciences are studies, where economics models are coupled to physical groundwater models to explore the economic implications of different groundwater management options (see also Section 3.2). Usually, those articles describe the individual models and technical details of coupling but the details of how the collaboration behind this was planned, conceptualized and performed are never mentioned. We assume that this information could be retrieved through a broader analysis of cited references, analyses of project reports (grey literature) and homepages–or, by asking the involved authors. This however, would mean a separate study with an entirely different scope and methodology which we decided not to include here.

In addition to the qualitative analysis of the contents, we searched the full texts of all analysed articles for occurrences of the terms “cross-”, multi-, “inter-”, transdisciplinary (in different word forms and spellings) and other terms related to integration, collaboration, etc. Only 15 of all analysed articles mentioned any such term in a related context. And, only 4 of these 15 articles mentioning these terms are not among those we identified as multi-disciplinary. Only one article [40] mentions in the introduction that the article is the result of an “interdisciplinary” study, but the remainder of the article does not elaborate on this.

We speculate that journal articles (short, concise, result-oriented) may not provide a suitable platform to describe the background and nature of multi- or interdisciplinary collaboration. We further speculate that the audience interested in this aspect of groundwater related research is just too small and authors do not want to use valuable space on aspects that may be regarded as outside the scope of the paper as such. Also, collaborative research may not be seen as a method worth describing as such.

### 3.7 Scientific impact of multidisciplinary articles

Table 14 shows the averaged impact factors of the journals for each article category. As impact factors are not always available for all journals in all periods, we have used the 2014 impact factor only, which may introduce a certain bias. Nevertheless, Table 14 hints that multidisciplinary research gets published in journals with lower impact factors and is much less cited than that in the natural science domain.

**Table 14:** Average journal impact factors (2014) in relation to the paper classifications.

	clearly natural sciences	most likely natural sciences	clearly social sciences	most likely social sciences	most likely multi-disciplinary	probably multi-disciplinary	maybe multi-disciplinary
Average Journal IF 2014	5.4	5.6	2.3	1.8	2.3	1.8	2.4

Whether interdisciplinary research gains more citations than disciplinary research is contentious [31]. Several authors have explored these questions and have arrived at different results (see discussion in e.g., [41, 42]). Personal experience and the present study’s results (see Table 15) clearly argue against the notion that multidisciplinary research across the natural-social sciences divide is highly cited, most likely due to the limited number of scientists working and interested in overlapping areas and thus citing their respective studies.

**Table 15:** Average citation numbers in relation to the paper classifications for the three analysed periods.

	Average of times cited							
	clearly natural sciences	most likely natural sciences	clearly social sciences	most likely social sciences	most likely multi-disciplinary	probably multi-disciplinary	maybe multi-disciplinary	Grand Total
1990–1994	86.9	19.9	29.6	17.3		7.5	22.4	45.1
2000–2004	87.8	57.0	22.3	10.0	12.0	2.0	11.0	49.2
2010–2014	25.8	26.8	8.5	5.0	7.0	4.8	3.0	17.9
Grand Total	47.7	31.0	16.6	8.8	8.0	5.1	11.0	30.3

### 3.8 Retrospective evaluation of results

The methods applied in this study and thus the corresponding results are based on several assumptions and include constraints in the sample selection and size. We review such assumptions and limitations and their implications for the results against the background of some observations we have made during the process.

In the three selected five-year intervals, approximately 16,000 journal articles with “groundwater” in their titles, distributed among well over 1400 journals, are listed in Scopus. In total, 3809 journal articles published over the 15-year period match the criteria we have used to identify the articles that potentially deal with a mix of natural and social science contents. Our sample contains 203 articles from 12 journals, representing 1.3% of all articles with “groundwater” in their titles and 5.3% of those matching our selection criteria. These percentages are admittedly small, and we may have missed journals that could have influenced the results. The small numbers are the results of our approach to rely on the tedious “manual” inspection of each article. Unfortunately, we are convinced that there is currently no alternative to manual inspection. The information provided by the Scopus and the WoS databases is not sufficiently complete (information on the disciplinary backgrounds of authors) and/or wrong or misleading (information on the disciplines of publications). Moreover, specific knowledge of the field is needed to make assumptions on the disciplinary classification of a paper, a citation or an author.

Apart from uncertainties potentially introduced through the sample size, we are also aware of some strong biases and unknown factors that our results may include. For example, some interesting journals did not yet exist in the earlier years of our analysis or have changed their names and foci throughout the years. Thus, some doubts remain concerning the validity of the results. We have therefore conducted some simple and quick “cross-validation” tests, looking at a number of randomly picked samples of articles published in the field of groundwater. For example, in the latest issues of *Groundwater* (http://onlinelibrary.wiley.com/doi/10.1111/gwat.2016.54.issue-4/issuetoc) and the *Hydrogeology Journal* (http://link.springer.com/journal/10040/24/6/page/1) (two major groundwater-related journals not included in our journal selection), 17 out of 17 and 17 out of 18 contributions, respectively, have a sole focus on natural science aspects of groundwater. The only report in the *Hydrogeology Journal* that deals with societal aspects of groundwater is written by a single author [43]and according to our indicators, has a mono-disciplinary focus on social sciences. Even the latest issue of *WRR (*http://link.springer.com/journal/11269/30/12/page/1), a journal where we had expected more social science or multidisciplinary content, is in line with our findings. The situation shown by these randomly picked examples is typical for the field; we invite the reader to perform their own tests.

In our attempt to measure the multidisciplinary collaboration between social and natural sciences in research, we have limited our analysis to articles in peer-reviewed international journals. This constraint is justified by the assumptions that journal articles constitute the most important forum for publishing research and that other forms of publications (web pages, project reports and so on) are quite inaccessible for systematic, quantitative analysis. However, some doubts remain whether journal literature adequately reflects the actual status. These uncertainties are confirmed by other authors [44–46], who claim that multidisciplinary activities are often too broad and complex to be published in journal articles. It also requires much more time to conduct and publish multidisciplinary research (e.g., [7, 47, 48]). Another reason noted by several authors is the lack of peer reviewers who are able to judge the quality of multidisciplinary research (see [2, 8, 49, 50]). Therefore, it is quite possible that journal articles, even if based on multidisciplinary projects, only present mono-disciplinary sections of them. This conclusion is also strongly supported by the results of our full text analysis (Section 3.6).

We have assumed that an article that is co-written by several authors with differing disciplinary orientations has the potential to be multidisciplinary. In hindsight, we assume this to be a reliable indicator, even if we have been surprised by some unexpected observations. An example is the high number of single authors dealing with aspects of groundwater at the interface between natural and social sciences and more generally, the involvement of multidisciplinary authors in the research carried out at the interface. The assumption that multidisciplinary research is carried out by two or more individuals from different disciplines is simplistic. It is rather a broad range of combinations, including individuals with multidisciplinary backgrounds who are involved. The interdisciplinarity of individuals plays a major role compared to interdisciplinarity involving two or more researchers. However, the question, whether an article written by a single author can be regarded as multidisciplinary is a tricky one. On the one hand, we found that articles by single authors dealing with a clearly multidisciplinary topic and citing a balanced mix of references from natural and social sciences, after all, focussed foremost on either natural or social sciences. For example, of two single authored articles combining groundwater and economic models in the widest sense, [51] has a strong focus on economics while [52] focuses on the natural science aspects. [53] on the other hand, while building heavily on the natural aspects of groundwater and surface water, finally focusses on law, governance and other “social” aspects. On the other hand, single authored papers may very well emerge from larger, interdisciplinary studies. Again, analysis beyond single articles is needed to understand the background and nature of a collaboration that may or not be behind the article.

Next, we have assumed that the cited references from both natural and social sciences indicate the potential to be multidisciplinary. We find this to be indeed a good indicator–it is not surprising that bibliometric analysis heavily relies on it. Unfortunately, we do not see an alternative to the tedious expert-based, manual inspection of all cited references, as the usually used WoS classifications should be very thoroughly cross-checked for accuracy (see Section 3.3.1).

Finally, we have assumed that the title of a publication can reveal if it is potentially multidisciplinary. We find this assumption rather weak but useful. As Table 16 shows, all of the papers classified as being most likely multidisciplinary also have titles indicating multidisciplinary research. On the other hand, of the papers finally classified as “clearly social sciences”, 71% have titles indicating multidisciplinary research, and even 21% of the papers identified as “clearly natural sciences” have titles indicating multidisciplinary research. Therefore, title assessment should not be used as an indicator of high weight.

**Table 16:** Comparison of a classification of the title only and the paper classification based on all indicators. Percentages show how many papers with titles indicating multidisciplinary research are finally classified as belonging to the individual categories.

	Paper classification						
Title indicating multidisciplinary contents	clearly natural sciences	most likely natural sciences	clearly social sciences	most likely social sciences	most likely multi-disciplinary	probably multi-disciplinary	maybe multi-disciplinary
yes	21.2%	43.8%	71.4%	55.6%	100.0%	66.7%	61.5%

While our study has targeted groundwater research, we still hope to find results that are representative of the collaboration between natural and social sciences on a more general level. Our sample does not allow any clear conclusions here. As pointed out in the introduction, groundwater–with its extraordinary importance for human lives and the environment and its manifold relations to almost every scientific discipline–should form an excellent subject for interdisciplinary research spanning natural and social sciences. We would even expect it to show a higher share of multidisciplinary collaboration between natural and social sciences than other fields of research. Thus, we are optimistic that our results could be representative. Of course, proofs would have to be provided by other experts carrying out similar studies in their respective fields of expertise. Having conducted this study, we are convinced that measuring interdisciplinarity between natural and social sciences requires a narrow field of research. Identifying interdisciplinarity necessitates knowledge and experience in the specific field, that is, knowing which topics, keywords and concepts indicate interdisciplinarity and possessing some sense of the different disciplines that may be involved. The experience of working and publishing in a multidisciplinary context is imperative as well.

### 3.9 Comparison with other authors’ findings

To our best knowledge, no published studies explicitly address the status of and developments in multidisciplinary collaboration between natural and social sciences in groundwater research or water research in general. While the groundwater community (e.g. [54–57]) and the hydrology community in general (e.g. [11, 58, 59]) argue strongly and convincingly for a more holistic perspective and more interdisciplinary approaches to hydrological research–in particular using collaboration with social sciences–no one seems to have thought about the three dimensions of interdisciplinarity described by [60], as follows: *what* is integrated, *how* it is done and *why* interdisciplinarity. In the broader context of hydrology [6, 12, 14] address the issue of collaboration between hydrology and social sciences, but their papers and those cited therein mainly list individual activities and achievements and do not reveal information that could be used to quantitatively assess the current status or past developments as a whole. However, a larger number of papers deal with the collaboration between natural and social sciences on a much broader level or use examples from other fields of science. It is outside the scope of this article to present a review of this literature, but we recommend the discussions presented by [2,7, 8, 61, 62, 63], who describe the various challenges and obstacles to performing multidisciplinary research. We find the relation between multidisciplinarity, on one hand, and career advancement and scientific reward structures for conducting multidisciplinary research, on the other hand, particularly interesting. It indicates that engaging in multidisciplinary research has a negative impact on individual careers, particularly of young researchers [38, 64–66]. To conclude, [4]mentions that the most common mistake is underestimating the depth of commitment and personal relationships needed for a successful interdisciplinary project.

## 4 Conclusions

In this study, we have aimed to answer the question of whether and how the frequently expressed demands for interdisciplinary collaboration between natural and social sciences are met by the scientific community. We have picked groundwater research as the case study because a) it has strong connections to almost all sectors of nature and society, and b) this is the field where we have the most insights and interest. The main conclusions drawn from this study are as follows:

### Share of journal articles based on multidisciplinary collaboration between natural and social sciences:

- Journal articles mention “interdisciplinarity” much more frequently now than 40 years ago, yet this increase is not reflected on a higher number of articles based on multidisciplinarity.
- Groundwater-related articles showing a high likelihood of resulting from collaborations between natural and social sciences occur in the 1% range, with a slightly increasing tendency.
- Multidisciplinary contents are published in social science or multidisciplinary-oriented journals rather than in journals focusing on water resources. The latter group increasingly concentrates on natural science aspects only.
- The impact factors of journals publishing multidisciplinary research contents are lower, and multidisciplinary articles receive considerably fewer citations.

### Who contributes articles based on multidisciplinary collaboration between natural and social sciences?

- The large majority of social scientists collaborating with natural scientists come from various fields of economic sciences, with the domination of agriculture-ecology-economy-groundwater and groundwater-economy-ecosystem services/environmental impact assessment subject areas.
- While mixed teams consisting of social scientists and natural scientists occur, individuals with multidisciplinary backgrounds or from disciplines located at the interface between social and natural sciences (geography and planning and management) have a large share.

### Measuring interdisciplinary research

- Measuring the degree of collaboration between natural and social sciences requires a detailed analysis of individual articles, their authors, keyword usage, references cited, combined with detailed knowledge of the field. On the other hand, measurement analysis based only on unsupervised bibliometric evaluation of large numbers of publications may yield strongly misleading results.

### Outlook and recommendations for future research

The study presented here provides some interesting insights into the development of the collaboration between natural and social sciences in the field of groundwater research. Despite the narrow field and the small sample size, we are confident that this study’s results are transferable to the collaboration between social and natural sciences in general. We had initially hoped to learn more about the nature of collaboration but have found that journal articles might be the wrong means to explore this. A truly interdisciplinary research endeavour must not necessarily result in interdisciplinary articles; length restrictions, disciplinary reviewers and authors’ career considerations may very well lead to the publication of small disciplinary snippets rather than the large picture. Moreover, we have scarcely found any description of applied interdisciplinary methodology (such as knowledge integration). Do authors not reflect on it, do they think it is not worth mentioning in the particular article, or is it just the lack of space that forces them to skip this? We therefore recommend and envision asking a sample of authors what they think of their own articles and our classifications, whether they would consider their works mono-disciplinary or interdisciplinary, and about the nature of the studies and projects from which their papers emerged.

## Supporting Information

S1 Spreadsheet Excel spreadsheet containing all the analysed articles together with the most relevant metadata, indicators, analysis results and classifications. (XLSX)

S1 Text Document listing the search string used to identify the articles analysed in this study. The search string combines the selected periods, journals, title words and keywords. It can be copied and pasted into the “advanced search” field at https://www.scopus.com/search/.
(DOCX)
