# Supplementary material for: Interdisciplinary Collaboration between Natural and Social Sciences – Status and Trends Exemplified in Groundwater Research
Source: PLoS One. 2017 Jan 27;12(1):e0170754. doi: 10.1371/journal.pone.0170754 (PMC5271333; doi:10.1371/journal.pone.0170754)
Supplement: S1 Text — The search string combines the selected periods, journals, title words and keywords. It can be copied and pasted into the “advanced search” field at https://www.scopus.com/search/. (DOCX) [file pone.0170754.s002.docx]

**S1 Text.** Search string to select all journal articles from the 12 selected journals from the periods 1990-1994, 2000-2004, 2010-2014 which have the terms “groundwater” or “ground water” in title (TI) or at least one of 60 “social science keywords” in keyword (KEY).

Note: the search string also lists the “social science keywords” used to determine potential multidisciplinary contents.

Several journal names give ambiguous results; therefore, it is better to search for ISSN numbers.

- Nature 0028-0836
- Nature Climate Change 1758-678X
- Nature Geoscience 1752-0894
- Science 0036-8075 and 1095-9203
- Sustainability 1937-0695
- Sustainability-Switzerland 2071-1050
- Water Resources Research 1944-7973 and 0043-1397

**Search string to be pasted in the advanced search field at** [**https://www.scopus.com/**](https://www.scopus.com/)

*SRCTYPE(j)*

*AND ((PUBYEAR  >  2009  AND  PUBYEAR  <  2015)*

*OR  (PUBYEAR  >  1989  AND  PUBYEAR  <  1995)*

*OR  (PUBYEAR  >  1999  AND  PUBYEAR  <  2005))*

*AND SRCTITLE("Water International")*

*OR SRCTITLE("Applied Geography")*

*OR SRCTITLE("Environmental Science & Policy")*

*OR SRCTITLE("Water Policy")*

*OR SRCTITLE("Ambio")*

*OR SRCTITLE("American Journal of Agricultural Economics")*

*OR SRCTITLE("Ecological Economics")*

*OR SRCTITLE("Journal of Environmental Planning and Management")*

*OR SRCTITLE("Journal of Water Resources Planning and Management")*

*OR ISSN(1944-7973) OR  ISSN ( 0043-1397 )*

*OR ISSN(0028-0836)*

*OR ISSN(1752-0894)*

*OR ISSN(1758-678X)*

*OR ISSN(0036-8075) or ISSN(1095-9203)*

*OR ISSN(1937-0695) OR ISSN(2071-1050)*

*AND TITLE (groundwater OR "ground water")*

*AND KEY("Stakeholder")*

*OR KEY("Adaptation")*

*OR KEY("Adaptive management")*

*OR KEY("Basin management")*

*OR KEY("Commerce")*

*OR KEY("Cost-benefit analysis")*

*OR KEY("Costs")*

*OR KEY("Decision making")*

*OR KEY("Decision support systems")*

*OR KEY("Developing country")*

*OR KEY("Developing world")*

*OR KEY("Economic and social effects")*

*OR KEY("Economic development")*

*OR KEY("Ecosystem service")*

*OR KEY("Environmental economics")*

*OR KEY("Environmental management")*

*OR KEY("Environmental policy")*

*OR KEY("Food security")*

*OR KEY("Governance approach")*

*OR KEY("Governance")*

*OR KEY("Human")*

*OR KEY("Hydroelectric power")*

*OR KEY("Integrated approach")*

*OR KEY("Integrated Water Resources Management")*

*OR KEY("Land use change")*

*OR KEY("legal ")*

*OR KEY("Management")*

*OR KEY("Natural resource")*

*OR KEY("Participatory approach")*

*OR KEY("Participatory")*

*OR KEY("Policy implementation")*

*OR KEY("Policy making")*

*OR KEY("Policy")*

*OR KEY("Population growth")*

*OR KEY("Public policy")*

*OR KEY("Regional planning")*

*OR KEY("Resource allocation")*

*OR KEY("Resource management")*

*OR KEY("Resource scarcity")*

*OR KEY("Risk assessment")*

*OR KEY("Sanitation")*

*OR KEY("Sustainability")*

*OR KEY("Sustainable development")*

*OR KEY("Urbanization")*

*OR KEY("Water availability")*

*OR KEY("Water conservation")*

*OR KEY("Water demand")*

*OR KEY("Water economics")*

*OR KEY("Water footprint")*

*OR KEY("Water governance")*

*OR KEY("Water law")*

*OR KEY("Water management")*

*OR KEY("Water planning")*

*OR KEY("Water policy")*

*OR KEY("Water resources development")*

*OR KEY("Water resources exploration")*

*OR KEY("Water resources management")*

*OR KEY("Water rights")*

*OR KEY("Water scarcity")*

*OR KEY("Water use efficiency")*

*OR KEY("Water use")*

**Searches used to retrieve the data shown in Fig 1**

*ALL ( "interdisciplinary" ) OR ALL ( "interdisciplinarity" ) OR ALL ( "inter-disciplinary" ) OR ALL ( "inter-disciplinarity" ) AND srctype(j)*

*TITLE-ABS-KEY ( groundwater OR "ground water" ) AND ALL ( "interdisciplinary" ) OR ALL ( "interdisciplinarity" ) OR ALL ( "inter-disciplinary" ) OR ALL ( "inter-disciplinarity" ) AND srctype(j)*
